# 3-(9-Anthrylmethyl)-1-benzylperimi­dinium hexa­fluoro­phosphate

**DOI:** 10.1107/S1600536809017140

**Published:** 2009-05-14

**Authors:** Gui-Yu Wang, Shi-Lu Zhang, Da-Bin Qin

**Affiliations:** a School of Chemistry and Chemical Engineering, China West Normal University, Nanchong 637002, People’s Republic of China

## Abstract

In the title compound, C_33_H_25_N_2_^+^·PF_6_^−^, the naphthalene ring system is twisted with respect to the anthracene and benzene rings, making dihedral angles of 72.40 (3) and 71.39 (4)°, respectively. The crystal structure is stabilized by intermolecular C—H⋯F hydrogen bonding. Four F atoms of the hexa­fluoro­phosphate anion are disordered over two sets of sites in a 0.645 (4):0.355 (4) ratio.

## Related literature

For the synthesis, see: Özdemir *et al.*. (2004 ▶); Aksenov *et al.* (2008 ▶). For related structures, see: Bazinet *et al.* (2007 ▶).
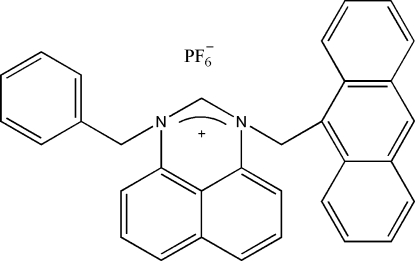


## Experimental

### 

#### Crystal data

C_33_H_25_N_2_^+^·F_6_P^−^
*M* *_r_* = 594.52
Triclinic, 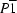

*a* = 9.9596 (13) Å
*b* = 11.8644 (17) Å
*c* = 11.8994 (12) Å
α = 94.436 (4)°
β = 95.159 (3)°
γ = 110.399 (6)°
*V* = 1303.6 (3) Å^3^
*Z* = 2
Mo *K*α radiation
μ = 0.18 mm^−1^
*T* = 113 K
0.22 × 0.20 × 0.18 mm

#### Data collection

Rigaku Saturn diffractometer
Absorption correction: none
9272 measured reflections
4823 independent reflections
3619 reflections with *I* > 2σ(*I*)
*R*_int_ = 0.035

#### Refinement

*R*[*F* ^2^ > 2σ(*F* ^2^)] = 0.042
*wR*(*F* ^2^) = 0.113
*S* = 1.01
4823 reflections
416 parameters
H-atom parameters constrained
Δρ_max_ = 0.20 e Å^−3^
Δρ_min_ = −0.49 e Å^−3^

### 

Data collection: *CrystalClear* (Rigaku/MSC, 2004 ▶); cell refinement: *CrystalClear*; data reduction: *CrystalClear*; program(s) used to solve structure: *SHELXS97* (Sheldrick, 2008 ▶); program(s) used to refine structure: *SHELXL97* (Sheldrick, 2008 ▶); molecular graphics: *ORTEP-3 for Windows* (Farrugia, 1997 ▶); software used to prepare material for publication: *CrystalStructure* (Rigaku/MSC, 2004 ▶).

## Supplementary Material

Crystal structure: contains datablocks global, I. DOI: 10.1107/S1600536809017140/xu2514sup1.cif

Structure factors: contains datablocks I. DOI: 10.1107/S1600536809017140/xu2514Isup2.hkl

Additional supplementary materials:  crystallographic information; 3D view; checkCIF report

## Figures and Tables

**Table 1:** Hydrogen-bond geometry (Å, °)

*D*—H⋯*A*	*D*—H	H⋯*A*	*D*⋯*A*	*D*—H⋯*A*
C10—H10⋯F5^i^	0.95	2.52	3.327 (3)	142
C11—H11⋯F2^i^	0.95	2.53	3.393 (4)	151
C19—H19*A*⋯F4^ii^	0.99	2.48	3.368 (3)	150
C27—H27⋯F1^iii^	0.95	2.47	3.401 (2)	165
C29—H29⋯F3^iii^	0.95	2.55	3.400 (3)	149

Symmetry codes: (i) 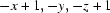; (ii) 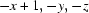; (iii) 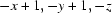.

**Fig. 1.**
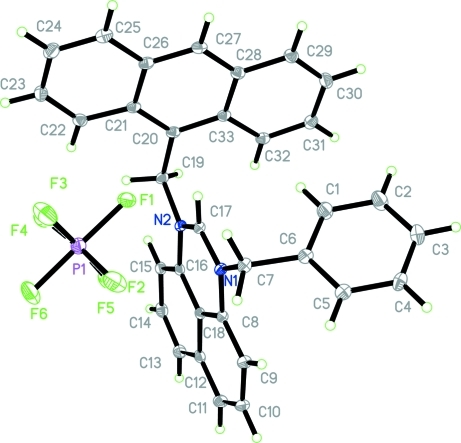
The molecular structure of the title compound, showing 30% probability displacement ellipsoids and the atomic numbering. The minor component of disordered PF6- has been omitted for clarity.

## supplementary crystallographic information

### Comment

In recent years, numerous cyclic N-heterocyclic carbene (NHC) precursors have been synthesized and structurally investigated. They have been recognized as powerfit ligands owing to their ability to coordinate very strongly to transition metals and main-group elements and an increasing use in organometallic chemistry, homogeneous catalysis. In addition, a number of biological activities of imidazolium salts have been reported including antimicrobial, antifungal, antitumor. We report here crystal structure of NHC precursor, the title compound.

The molecular structure is shown in Fig. 1. The naphthalene ring system is twisted with the anthracene ring and benzene ring with the dihedral angles of 72.40 (3)° and 71.39 (4)°. The crystal structure is stabilized by C—H···F hydrogen bonds (Table 1).

### Experimental

The title compound was prepared according to the procedure reported by Özdemir *et al.* (2004) and Aksenov *et al.* (2008). Yellow single crystals suitable for X-ray diffraction were obtained by recrystallization from acetonitrile.

### Refinement

H atoms were placed in calculated positions with C—H = 0.95–0.99 Å, and refined in riding mode with *U*_iso_(H) = 1.2*U*_eq_(C). The four equatorial F atoms (F_2_–F_5_) in the anion PF_6_ were disordered at two palces, occupancies were refined to a 0.645 (4):0.355 (4) ratio.

### Crystal data

**Table:**

C_33_H_25_N_2_^+^·F_6_P^−^	*Z* = 2
*M**_r_* = 594.52	*F*(000) = 612
Triclinic, *P*1	*D*_x_ = 1.515 Mg m^−^^3^
Hall symbol: -P 1	Mo *K*α radiation, λ = 0.71070 Å
*a* = 9.9596 (13) Å	Cell parameters from 3227 reflections
*b* = 11.8644 (17) Å	θ = 2.2–28.0°
*c* = 11.8994 (12) Å	µ = 0.18 mm^−^^1^
α = 94.436 (4)°	*T* = 113 K
β = 95.159 (3)°	Prism, yellow
γ = 110.399 (6)°	0.22 × 0.20 × 0.18 mm
*V* = 1303.6 (3) Å^3^	

### Data collection

**Table:**

Rigaku Saturn diffractometer	3619 reflections with *I* > 2σ(*I*)
Radiation source: fine-focus sealed tube	*R*_int_ = 0.035
graphite	θ_max_ = 25.5°, θ_min_ = 2.2°
Detector resolution: 7.31 pixels mm^-1^	*h* = −12→12
ω scans	*k* = −14→13
9272 measured reflections	*l* = −14→14
4823 independent reflections	

### Refinement

**Table:**

Refinement on *F*^2^	Primary atom site location: structure-invariant direct methods
Least-squares matrix: full	Secondary atom site location: difference Fourier map
*R*[*F*^2^ > 2σ(*F*^2^)] = 0.042	Hydrogen site location: inferred from neighbouring sites
*wR*(*F*^2^) = 0.113	H-atom parameters constrained
*S* = 1.01	*w* = 1/[σ^2^(*F*_o_^2^) + (0.0672*P*)^2^] where *P* = (*F*_o_^2^ + 2*F*_c_^2^)/3
4823 reflections	(Δ/σ)_max_ < 0.001
416 parameters	Δρ_max_ = 0.20 e Å^−^^3^
0 restraints	Δρ_min_ = −0.49 e Å^−^^3^

### Special details

**Table:**

Geometry. All esds (except the esd in the dihedral angle between two l.s. planes) are estimated using the full covariance matrix. The cell esds are taken into account individually in the estimation of esds in distances, angles and torsion angles; correlations between esds in cell parameters are only used when they are defined by crystal symmetry. An approximate (isotropic) treatment of cell esds is used for estimating esds involving l.s. planes.
Refinement. Refinement of *F*^2^ against ALL reflections. The weighted *R*-factor *wR* and goodness of fit *S* are based on *F*^2^, conventional *R*-factors *R* are based on *F*, with *F* set to zero for negative *F*^2^. The threshold expression of *F*^2^ > σ(*F*^2^) is used only for calculating *R*-factors(gt) *etc*. and is not relevant to the choice of reflections for refinement. *R*-factors based on *F*^2^ are statistically about twice as large as those based on *F*, and *R*- factors based on ALL data will be even larger.

### Fractional atomic coordinates and isotropic or equivalent isotropic displacement parameters (Å^2^)

**Table:**

	*x*	*y*	*z*	*U*_iso_*/*U*_eq_	Occ. (<1)
N1	0.65525 (14)	0.26739 (12)	0.34502 (11)	0.0171 (3)	
N2	0.76633 (15)	0.21452 (12)	0.19570 (11)	0.0166 (3)	
C1	0.7929 (2)	0.54158 (17)	0.41449 (17)	0.0318 (5)	
H1	0.8104	0.5239	0.3391	0.038*	
C2	0.8809 (2)	0.64882 (17)	0.47870 (18)	0.0370 (5)	
H2	0.9593	0.7036	0.4478	0.044*	
C3	0.8547 (2)	0.67608 (17)	0.58745 (17)	0.0313 (5)	
H3	0.9134	0.7505	0.6308	0.038*	
C4	0.7426 (2)	0.59450 (16)	0.63322 (16)	0.0272 (4)	
H4	0.7254	0.6122	0.7087	0.033*	
C5	0.65546 (19)	0.48705 (16)	0.56896 (15)	0.0243 (4)	
H5	0.5785	0.4316	0.6007	0.029*	
C6	0.67932 (18)	0.45972 (15)	0.45940 (15)	0.0209 (4)	
C7	0.57901 (18)	0.34585 (15)	0.38640 (15)	0.0209 (4)	
H7A	0.5035	0.2998	0.4312	0.025*	
H7B	0.5306	0.3689	0.3204	0.025*	
C8	0.68755 (18)	0.18549 (14)	0.41469 (14)	0.0182 (4)	
C9	0.64948 (18)	0.17407 (15)	0.52252 (14)	0.0230 (4)	
H9	0.6024	0.2235	0.5550	0.028*	
C10	0.68127 (19)	0.08799 (16)	0.58429 (16)	0.0264 (4)	
H10	0.6532	0.0783	0.6582	0.032*	
C11	0.75186 (19)	0.01810 (16)	0.53968 (16)	0.0266 (4)	
H11	0.7727	−0.0389	0.5832	0.032*	
C12	0.79414 (19)	0.02947 (15)	0.43000 (15)	0.0223 (4)	
C13	0.86924 (19)	−0.03935 (15)	0.38004 (16)	0.0254 (4)	
H13	0.8926	−0.0969	0.4213	0.030*	
C14	0.90839 (19)	−0.02426 (15)	0.27382 (16)	0.0250 (4)	
H14	0.9593	−0.0712	0.2427	0.030*	
C15	0.87524 (18)	0.05926 (14)	0.20896 (15)	0.0203 (4)	
H15	0.9025	0.0684	0.1348	0.024*	
C16	0.80270 (17)	0.12715 (14)	0.25529 (14)	0.0171 (4)	
C17	0.69340 (17)	0.27509 (14)	0.24203 (14)	0.0168 (4)	
H17	0.6660	0.3287	0.1978	0.020*	
C18	0.76120 (17)	0.11481 (14)	0.36533 (14)	0.0177 (4)	
C19	0.81402 (19)	0.23771 (15)	0.08085 (14)	0.0204 (4)	
H19A	0.7622	0.1649	0.0260	0.024*	
H19B	0.9186	0.2525	0.0851	0.024*	
C20	0.78569 (18)	0.34486 (15)	0.03857 (14)	0.0189 (4)	
C21	0.67085 (18)	0.32839 (15)	−0.04710 (14)	0.0198 (4)	
C22	0.58415 (19)	0.21358 (15)	−0.10874 (15)	0.0232 (4)	
H22	0.6033	0.1432	−0.0922	0.028*	
C23	0.4749 (2)	0.20365 (16)	−0.19052 (15)	0.0276 (4)	
H23	0.4210	0.1267	−0.2316	0.033*	
C24	0.4395 (2)	0.30512 (17)	−0.21595 (16)	0.0295 (4)	
H24	0.3605	0.2955	−0.2716	0.035*	
C25	0.51861 (19)	0.41642 (17)	−0.16064 (15)	0.0265 (4)	
H25	0.4944	0.4843	−0.1780	0.032*	
C26	0.63810 (19)	0.43274 (15)	−0.07648 (14)	0.0205 (4)	
C27	0.72088 (18)	0.54694 (15)	−0.02117 (14)	0.0221 (4)	
H27	0.6969	0.6148	−0.0393	0.026*	
C28	0.83807 (18)	0.56432 (15)	0.06025 (14)	0.0204 (4)	
C29	0.92430 (19)	0.68278 (15)	0.11489 (15)	0.0244 (4)	
H29	0.9021	0.7508	0.0948	0.029*	
C30	1.0374 (2)	0.69881 (16)	0.19508 (16)	0.0264 (4)	
H30	1.0940	0.7779	0.2304	0.032*	
C31	1.07145 (19)	0.59810 (16)	0.22627 (15)	0.0249 (4)	
H31	1.1508	0.6104	0.2825	0.030*	
C32	0.99184 (18)	0.48391 (15)	0.17669 (14)	0.0208 (4)	
H32	1.0161	0.4177	0.1994	0.025*	
C33	0.87223 (18)	0.46190 (15)	0.09092 (14)	0.0192 (4)	
F1	0.40915 (11)	0.24779 (9)	0.12667 (9)	0.0327 (3)	
F2	0.2510 (4)	0.1531 (2)	0.2443 (3)	0.0547 (10)	0.645 (4)
F3	0.1733 (2)	0.1364 (2)	0.0581 (3)	0.0549 (11)	0.645 (4)
F4	0.3399 (3)	0.05825 (18)	0.01761 (18)	0.0455 (8)	0.645 (4)
F5	0.4188 (2)	0.07577 (17)	0.2026 (2)	0.0432 (8)	0.645 (4)
F2'	0.3311 (7)	0.1323 (6)	0.2590 (4)	0.075 (3)	0.355 (4)
F3'	0.1746 (4)	0.1825 (3)	0.1473 (5)	0.0555 (17)	0.355 (4)
F4'	0.2570 (6)	0.1275 (4)	−0.0041 (3)	0.066 (2)	0.355 (4)
F5'	0.4147 (6)	0.0736 (4)	0.1032 (8)	0.087 (3)	0.355 (4)
F6	0.18320 (13)	−0.02144 (10)	0.13375 (11)	0.0468 (3)	
P1	0.29507 (5)	0.11278 (4)	0.12934 (4)	0.02730 (16)	

### Atomic displacement parameters (Å^2^)

**Table:**

	*U*^11^	*U*^22^	*U*^33^	*U*^12^	*U*^13^	*U*^23^
N1	0.0184 (7)	0.0182 (7)	0.0149 (8)	0.0068 (6)	0.0022 (6)	0.0015 (6)
N2	0.0189 (7)	0.0162 (7)	0.0146 (8)	0.0065 (6)	0.0007 (6)	0.0022 (5)
C1	0.0358 (11)	0.0290 (11)	0.0294 (11)	0.0094 (9)	0.0118 (9)	−0.0018 (8)
C2	0.0348 (12)	0.0277 (11)	0.0437 (14)	0.0042 (9)	0.0139 (10)	−0.0022 (9)
C3	0.0293 (11)	0.0254 (10)	0.0358 (12)	0.0089 (8)	−0.0007 (9)	−0.0068 (8)
C4	0.0307 (11)	0.0321 (11)	0.0208 (10)	0.0162 (8)	−0.0003 (8)	−0.0044 (8)
C5	0.0252 (10)	0.0271 (10)	0.0236 (10)	0.0129 (8)	0.0031 (8)	0.0030 (7)
C6	0.0220 (9)	0.0238 (9)	0.0207 (10)	0.0129 (7)	0.0035 (7)	0.0024 (7)
C7	0.0216 (9)	0.0240 (9)	0.0200 (10)	0.0119 (7)	0.0034 (7)	0.0013 (7)
C8	0.0180 (9)	0.0176 (9)	0.0161 (9)	0.0033 (7)	−0.0022 (7)	0.0036 (7)
C9	0.0241 (10)	0.0243 (10)	0.0199 (10)	0.0074 (8)	0.0033 (8)	0.0041 (7)
C10	0.0275 (10)	0.0285 (10)	0.0180 (10)	0.0024 (8)	0.0025 (8)	0.0078 (7)
C11	0.0271 (10)	0.0245 (10)	0.0254 (11)	0.0060 (8)	−0.0033 (8)	0.0096 (8)
C12	0.0216 (9)	0.0166 (9)	0.0234 (10)	0.0020 (7)	−0.0052 (7)	0.0035 (7)
C13	0.0280 (10)	0.0199 (9)	0.0280 (11)	0.0101 (8)	−0.0058 (8)	0.0046 (7)
C14	0.0251 (10)	0.0184 (9)	0.0317 (11)	0.0100 (8)	−0.0018 (8)	−0.0001 (7)
C15	0.0215 (9)	0.0173 (9)	0.0203 (10)	0.0058 (7)	0.0001 (7)	0.0000 (7)
C16	0.0154 (8)	0.0145 (8)	0.0185 (9)	0.0031 (6)	−0.0031 (7)	0.0020 (6)
C17	0.0171 (9)	0.0174 (8)	0.0152 (9)	0.0057 (7)	0.0001 (7)	0.0023 (6)
C18	0.0152 (8)	0.0150 (8)	0.0185 (9)	0.0016 (6)	−0.0032 (7)	0.0005 (7)
C19	0.0273 (10)	0.0218 (9)	0.0148 (9)	0.0113 (7)	0.0053 (7)	0.0029 (7)
C20	0.0241 (9)	0.0204 (9)	0.0156 (9)	0.0105 (7)	0.0080 (7)	0.0040 (7)
C21	0.0230 (9)	0.0228 (9)	0.0157 (9)	0.0086 (7)	0.0080 (7)	0.0060 (7)
C22	0.0281 (10)	0.0210 (9)	0.0200 (10)	0.0068 (7)	0.0080 (8)	0.0039 (7)
C23	0.0279 (10)	0.0262 (10)	0.0225 (10)	0.0026 (8)	0.0030 (8)	0.0001 (8)
C24	0.0246 (10)	0.0376 (11)	0.0235 (11)	0.0076 (8)	0.0001 (8)	0.0059 (8)
C25	0.0267 (10)	0.0318 (11)	0.0239 (10)	0.0127 (8)	0.0052 (8)	0.0088 (8)
C26	0.0235 (9)	0.0246 (9)	0.0160 (9)	0.0104 (7)	0.0072 (7)	0.0049 (7)
C27	0.0285 (10)	0.0231 (9)	0.0199 (10)	0.0139 (8)	0.0068 (8)	0.0073 (7)
C28	0.0230 (9)	0.0206 (9)	0.0199 (10)	0.0094 (7)	0.0059 (7)	0.0040 (7)
C29	0.0314 (10)	0.0200 (9)	0.0236 (10)	0.0109 (8)	0.0065 (8)	0.0028 (7)
C30	0.0289 (10)	0.0207 (10)	0.0267 (11)	0.0056 (8)	0.0052 (8)	−0.0013 (7)
C31	0.0230 (9)	0.0294 (10)	0.0211 (10)	0.0080 (8)	0.0031 (7)	0.0024 (8)
C32	0.0238 (9)	0.0227 (9)	0.0191 (10)	0.0108 (7)	0.0062 (7)	0.0058 (7)
C33	0.0222 (9)	0.0224 (9)	0.0153 (9)	0.0091 (7)	0.0070 (7)	0.0046 (7)
F1	0.0280 (6)	0.0320 (6)	0.0335 (7)	0.0037 (5)	0.0002 (5)	0.0139 (5)
F2	0.085 (2)	0.0287 (12)	0.047 (2)	0.0098 (14)	0.0414 (19)	−0.0042 (11)
F3	0.0300 (12)	0.0377 (13)	0.089 (3)	0.0041 (10)	−0.0160 (14)	0.0271 (14)
F4	0.0612 (18)	0.0338 (12)	0.0269 (12)	−0.0031 (11)	0.0200 (11)	−0.0041 (9)
F5	0.0394 (13)	0.0247 (10)	0.0576 (17)	0.0043 (8)	−0.0149 (12)	0.0163 (10)
F2'	0.083 (5)	0.074 (4)	0.029 (3)	−0.024 (3)	−0.008 (3)	0.031 (3)
F3'	0.030 (2)	0.031 (2)	0.099 (5)	0.0025 (16)	0.022 (2)	−0.005 (2)
F4'	0.072 (4)	0.065 (3)	0.026 (2)	−0.016 (3)	0.000 (2)	−0.0096 (19)
F5'	0.062 (3)	0.031 (2)	0.192 (10)	0.033 (2)	0.064 (5)	0.029 (4)
F6	0.0406 (7)	0.0306 (7)	0.0563 (9)	−0.0037 (5)	0.0159 (6)	−0.0043 (6)
P1	0.0263 (3)	0.0282 (3)	0.0224 (3)	0.0036 (2)	0.0047 (2)	0.0008 (2)

### Geometric parameters (Å, °)

**Table:**

N1—C17	1.316 (2)	C19—C20	1.507 (2)
N1—C8	1.425 (2)	C19—H19A	0.9900
N1—C7	1.475 (2)	C19—H19B	0.9900
N2—C17	1.312 (2)	C20—C21	1.410 (2)
N2—C16	1.428 (2)	C20—C33	1.412 (2)
N2—C19	1.501 (2)	C21—C22	1.432 (2)
C1—C2	1.387 (3)	C21—C26	1.445 (2)
C1—C6	1.390 (3)	C22—C23	1.359 (2)
C1—H1	0.9500	C22—H22	0.9500
C2—C3	1.379 (3)	C23—C24	1.414 (3)
C2—H2	0.9500	C23—H23	0.9500
C3—C4	1.385 (3)	C24—C25	1.358 (2)
C3—H3	0.9500	C24—H24	0.9500
C4—C5	1.386 (2)	C25—C26	1.432 (2)
C4—H4	0.9500	C25—H25	0.9500
C5—C6	1.380 (2)	C26—C27	1.391 (2)
C5—H5	0.9500	C27—C28	1.393 (2)
C6—C7	1.516 (2)	C27—H27	0.9500
C7—H7A	0.9900	C28—C29	1.433 (2)
C7—H7B	0.9900	C28—C33	1.435 (2)
C8—C9	1.374 (2)	C29—C30	1.359 (3)
C8—C18	1.420 (2)	C29—H29	0.9500
C9—C10	1.408 (2)	C30—C31	1.417 (2)
C9—H9	0.9500	C30—H30	0.9500
C10—C11	1.367 (3)	C31—C32	1.362 (2)
C10—H10	0.9500	C31—H31	0.9500
C11—C12	1.409 (3)	C32—C33	1.433 (2)
C11—H11	0.9500	C32—H32	0.9500
C12—C13	1.418 (3)	F1—P1	1.6135 (11)
C12—C18	1.425 (2)	F2—P1	1.564 (2)
C13—C14	1.363 (3)	F3—P1	1.5353 (19)
C13—H13	0.9500	F4—P1	1.600 (2)
C14—C15	1.410 (2)	F5—P1	1.6477 (19)
C14—H14	0.9500	F2'—P1	1.533 (5)
C15—C16	1.372 (2)	F3'—P1	1.697 (4)
C15—H15	0.9500	F4'—P1	1.636 (4)
C16—C18	1.411 (2)	F5'—P1	1.471 (4)
C17—H17	0.9500	F6—P1	1.6021 (12)
			
C17—N1—C8	120.62 (14)	C22—C23—C24	121.57 (17)
C17—N1—C7	118.36 (14)	C22—C23—H23	119.2
C8—N1—C7	121.02 (14)	C24—C23—H23	119.2
C17—N2—C16	120.01 (14)	C25—C24—C23	119.87 (18)
C17—N2—C19	120.46 (14)	C25—C24—H24	120.1
C16—N2—C19	119.52 (13)	C23—C24—H24	120.1
C2—C1—C6	120.56 (18)	C24—C25—C26	120.87 (17)
C2—C1—H1	119.7	C24—C25—H25	119.6
C6—C1—H1	119.7	C26—C25—H25	119.6
C3—C2—C1	120.07 (18)	C27—C26—C25	120.98 (16)
C3—C2—H2	120.0	C27—C26—C21	119.68 (16)
C1—C2—H2	120.0	C25—C26—C21	119.32 (16)
C2—C3—C4	119.72 (17)	C26—C27—C28	121.61 (16)
C2—C3—H3	120.1	C26—C27—H27	119.2
C4—C3—H3	120.1	C28—C27—H27	119.2
C3—C4—C5	119.99 (18)	C27—C28—C29	121.05 (16)
C3—C4—H4	120.0	C27—C28—C33	119.59 (15)
C5—C4—H4	120.0	C29—C28—C33	119.36 (16)
C6—C5—C4	120.76 (17)	C30—C29—C28	120.69 (16)
C6—C5—H5	119.6	C30—C29—H29	119.7
C4—C5—H5	119.6	C28—C29—H29	119.7
C5—C6—C1	118.88 (16)	C29—C30—C31	120.31 (16)
C5—C6—C7	120.93 (16)	C29—C30—H30	119.8
C1—C6—C7	120.11 (16)	C31—C30—H30	119.8
N1—C7—C6	112.65 (13)	C32—C31—C30	120.85 (17)
N1—C7—H7A	109.1	C32—C31—H31	119.6
C6—C7—H7A	109.1	C30—C31—H31	119.6
N1—C7—H7B	109.1	C31—C32—C33	121.20 (16)
C6—C7—H7B	109.1	C31—C32—H32	119.4
H7A—C7—H7B	107.8	C33—C32—H32	119.4
C9—C8—C18	121.59 (16)	C20—C33—C32	123.01 (16)
C9—C8—N1	122.67 (15)	C20—C33—C28	119.38 (16)
C18—C8—N1	115.74 (15)	C32—C33—C28	117.59 (15)
C8—C9—C10	119.04 (17)	F5'—P1—F2'	97.4 (5)
C8—C9—H9	120.5	F5'—P1—F3	134.8 (4)
C10—C9—H9	120.5	F2'—P1—F3	127.7 (3)
C11—C10—C9	121.11 (17)	F5'—P1—F2	132.0 (4)
C11—C10—H10	119.4	F2'—P1—F2	34.6 (3)
C9—C10—H10	119.4	F3—P1—F2	93.12 (18)
C10—C11—C12	120.93 (17)	F5'—P1—F4	44.5 (3)
C10—C11—H11	119.5	F2'—P1—F4	141.3 (4)
C12—C11—H11	119.5	F3—P1—F4	90.88 (17)
C11—C12—C13	123.40 (16)	F2—P1—F4	173.66 (14)
C11—C12—C18	118.90 (16)	F5'—P1—F6	94.76 (17)
C13—C12—C18	117.71 (16)	F2'—P1—F6	91.31 (19)
C14—C13—C12	121.02 (16)	F3—P1—F6	87.39 (9)
C14—C13—H13	119.5	F2—P1—F6	88.91 (11)
C12—C13—H13	119.5	F4—P1—F6	86.36 (8)
C13—C14—C15	121.62 (17)	F5'—P1—F1	84.92 (17)
C13—C14—H14	119.2	F2'—P1—F1	87.91 (19)
C15—C14—H14	119.2	F3—P1—F1	93.42 (9)
C16—C15—C14	118.59 (17)	F2—P1—F1	90.69 (11)
C16—C15—H15	120.7	F4—P1—F1	93.98 (8)
C14—C15—H15	120.7	F6—P1—F1	179.11 (7)
C15—C16—C18	121.50 (16)	F5'—P1—F4'	91.6 (4)
C15—C16—N2	122.08 (16)	F2'—P1—F4'	165.3 (3)
C18—C16—N2	116.42 (15)	F3—P1—F4'	43.9 (2)
N2—C17—N1	125.09 (15)	F2—P1—F4'	135.0 (3)
N2—C17—H17	117.5	F4—P1—F4'	50.3 (2)
N1—C17—H17	117.5	F6—P1—F4'	99.54 (15)
C16—C18—C8	122.03 (15)	F1—P1—F4'	81.30 (15)
C16—C18—C12	119.56 (16)	F5'—P1—F5	43.9 (3)
C8—C18—C12	118.41 (16)	F2'—P1—F5	54.1 (3)
N2—C19—C20	112.12 (13)	F3—P1—F5	175.39 (11)
N2—C19—H19A	109.2	F2—P1—F5	88.54 (16)
C20—C19—H19A	109.2	F4—P1—F5	87.11 (15)
N2—C19—H19B	109.2	F6—P1—F5	88.35 (9)
C20—C19—H19B	109.2	F1—P1—F5	90.85 (8)
H19A—C19—H19B	107.9	F4'—P1—F5	135.5 (3)
C21—C20—C33	120.73 (15)	F5'—P1—F3'	168.7 (2)
C21—C20—C19	120.84 (15)	F2'—P1—F3'	86.9 (3)
C33—C20—C19	118.35 (15)	F3—P1—F3'	41.43 (18)
C20—C21—C22	123.91 (16)	F2—P1—F3'	52.9 (2)
C20—C21—C26	118.90 (15)	F4—P1—F3'	131.8 (2)
C22—C21—C26	117.19 (16)	F6—P1—F3'	95.57 (13)
C23—C22—C21	121.10 (17)	F1—P1—F3'	84.82 (13)
C23—C22—H22	119.5	F4'—P1—F3'	82.2 (3)
C21—C22—H22	119.5	F5—P1—F3'	141.0 (2)
			
C6—C1—C2—C3	−1.1 (3)	N1—C8—C18—C12	−178.82 (14)
C1—C2—C3—C4	1.7 (3)	C11—C12—C18—C16	−179.93 (15)
C2—C3—C4—C5	−1.3 (3)	C13—C12—C18—C16	0.5 (2)
C3—C4—C5—C6	0.3 (3)	C11—C12—C18—C8	0.3 (2)
C4—C5—C6—C1	0.3 (3)	C13—C12—C18—C8	−179.26 (14)
C4—C5—C6—C7	−176.36 (16)	C17—N2—C19—C20	7.6 (2)
C2—C1—C6—C5	0.1 (3)	C16—N2—C19—C20	−171.50 (14)
C2—C1—C6—C7	176.79 (17)	N2—C19—C20—C21	−104.92 (18)
C17—N1—C7—C6	−97.27 (17)	N2—C19—C20—C33	71.8 (2)
C8—N1—C7—C6	82.48 (19)	C33—C20—C21—C22	176.07 (16)
C5—C6—C7—N1	−123.00 (17)	C19—C20—C21—C22	−7.2 (3)
C1—C6—C7—N1	60.4 (2)	C33—C20—C21—C26	−3.2 (2)
C17—N1—C8—C9	−179.79 (15)	C19—C20—C21—C26	173.47 (15)
C7—N1—C8—C9	0.5 (2)	C20—C21—C22—C23	−179.96 (17)
C17—N1—C8—C18	−0.3 (2)	C26—C21—C22—C23	−0.7 (3)
C7—N1—C8—C18	179.97 (13)	C21—C22—C23—C24	−1.8 (3)
C18—C8—C9—C10	−1.6 (3)	C22—C23—C24—C25	2.2 (3)
N1—C8—C9—C10	177.89 (15)	C23—C24—C25—C26	0.0 (3)
C8—C9—C10—C11	1.5 (3)	C24—C25—C26—C27	178.84 (17)
C9—C10—C11—C12	−0.5 (3)	C24—C25—C26—C21	−2.5 (3)
C10—C11—C12—C13	179.12 (16)	C20—C21—C26—C27	0.8 (2)
C10—C11—C12—C18	−0.4 (3)	C22—C21—C26—C27	−178.56 (15)
C11—C12—C13—C14	−179.60 (16)	C20—C21—C26—C25	−177.89 (15)
C18—C12—C13—C14	0.0 (2)	C22—C21—C26—C25	2.8 (2)
C12—C13—C14—C15	−0.5 (3)	C25—C26—C27—C28	−179.52 (16)
C13—C14—C15—C16	0.5 (3)	C21—C26—C27—C28	1.8 (3)
C14—C15—C16—C18	0.0 (2)	C26—C27—C28—C29	178.68 (16)
C14—C15—C16—N2	179.37 (15)	C26—C27—C28—C33	−2.0 (3)
C17—N2—C16—C15	178.13 (14)	C27—C28—C29—C30	179.29 (17)
C19—N2—C16—C15	−2.8 (2)	C33—C28—C29—C30	0.0 (3)
C17—N2—C16—C18	−2.4 (2)	C28—C29—C30—C31	−0.3 (3)
C19—N2—C16—C18	176.65 (13)	C29—C30—C31—C32	0.0 (3)
C16—N2—C17—N1	3.9 (2)	C30—C31—C32—C33	0.6 (3)
C19—N2—C17—N1	−175.18 (14)	C21—C20—C33—C32	−178.74 (16)
C8—N1—C17—N2	−2.5 (2)	C19—C20—C33—C32	4.5 (3)
C7—N1—C17—N2	177.28 (14)	C21—C20—C33—C28	3.1 (3)
C15—C16—C18—C8	179.28 (15)	C19—C20—C33—C28	−173.69 (15)
N2—C16—C18—C8	−0.2 (2)	C31—C32—C33—C20	−179.07 (17)
C15—C16—C18—C12	−0.4 (2)	C31—C32—C33—C28	−0.8 (3)
N2—C16—C18—C12	−179.89 (14)	C27—C28—C33—C20	−0.5 (3)
C9—C8—C18—C16	−179.03 (15)	C29—C28—C33—C20	178.87 (15)
N1—C8—C18—C16	1.5 (2)	C27—C28—C33—C32	−178.75 (15)
C9—C8—C18—C12	0.7 (2)	C29—C28—C33—C32	0.6 (2)

### Hydrogen-bond geometry (Å, °)

**Table:**

*D*—H···*A*	*D*—H	H···*A*	*D*···*A*	*D*—H···*A*
C10—H10···F5^i^	0.95	2.52	3.327 (3)	142
C11—H11···F2^i^	0.95	2.53	3.393 (4)	151
C19—H19A···F4^ii^	0.99	2.48	3.368 (3)	150
C27—H27···F1^iii^	0.95	2.47	3.401 (2)	165
C29—H29···F3^iii^	0.95	2.55	3.400 (3)	149

Symmetry codes: (i) −*x*+1, −*y*, −*z*+1; (ii) −*x*+1, −*y*, −*z*; (iii) −*x*+1, −*y*+1, −*z*.
